# Distribution- and Metabolism-Based Drug Discovery: A Potassium-Competitive Acid Blocker as a Proof of Concept

**DOI:** 10.34133/2022/9852518

**Published:** 2022-07-22

**Authors:** Ming-Shu Wang, Yi Gong, Lin-Sheng Zhuo, Xing-Xing Shi, Yan-Guang Tian, Chang-Kang Huang, Wei Huang, Guang-Fu Yang

**Affiliations:** ^1^Key Laboratory of Pesticide & Chemical Biology of Ministry of Education, International Joint Research Center for Intelligent Biosensor Technology and Health, College of Chemistry, Central China Normal University, Wuhan 430079, China; ^2^Nanjing Shuohui Pharmatechnology Co., Ltd., Nanjing 210046, China

## Abstract

Conventional methods of drug design require compromise in the form of side effects to achieve sufficient efficacy because targeting drugs to specific organs remains challenging. Thus, new strategies to design organ-specific drugs that induce little toxicity are needed. Based on characteristic tissue niche-mediated drug distribution (TNMDD) and patterns of drug metabolism into specific intermediates, we propose a strategy of distribution- and metabolism-based drug design (DMBDD); through a physicochemical property-driven distribution optimization cooperated with a well-designed metabolism pathway, SH-337, a candidate potassium-competitive acid blocker (P-CAB), was designed. SH-337 showed specific distribution in the stomach in the long term and was rapidly cleared from the systemic compartment. Therefore, SH-337 exerted a comparable pharmacological effect but a 3.3-fold higher no observed adverse effect level (NOAEL) compared with FDA-approved vonoprazan. This study contributes a proof-of-concept demonstration of DMBDD and provides a new perspective for the development of highly efficient, organ-specific drugs with low toxicity.

## 1. Introduction

Reduced productivity is one of the greatest challenges that the pharmaceutical industry faces today [1–5]. In the early days of drug discovery, poor efficacy caused by nonoptimal pharmacokinetics and low bioavailability were thought to be the main reasons that drug candidates failed to reach the market [6–8]. Therefore, traditional pharmacochemical patterns reflected a commitment to pursuing compounds with low clearance and high systemic exposure to ensure effectiveness [7, 9–12]. However, increased development-limiting toxicity has been consistently observed, despite high systemic exposure. The attrition of approximately 30% of drug candidates in development and withdrawal of drugs can be attributed to safety issues [13–20]. Therefore, many drugs are still produced based on a compromise between their side effects and efficacy.

Because most diseases are organ localized, high systemic exposure to drugs and their specific metabolites can result in unrelated and undesired effects at nontarget sites, leading to drug-induced toxicity [16, 21–23]. Therefore, the design of drugs with an organ-specific distribution is a smart strategy to improve efficacy while reducing side effects. Soft drugs (SDs), which are rapidly metabolized to inactive metabolites after exerting their therapeutic effects, were first introduced for therapeutic applications in which the desired activity is localized, short, or ultrashort [24]. These drugs are mainly directed to tissues and organs, such as the eye, skin, intestines, nasal mucosa, or lungs, and can be directly administered through titration, smearing, intestinal deposits, or inhalation but are rarely applied directly to organs such as the stomach, heart, and brain, which are primarily reached through systemic circulation [24]. Recent advancements in drug delivery technology have made it possible to enhance the delivery of a therapeutic to its target organ, minimizing off-target drug accumulation and improving patient compliance [25]. For instance, antibody–drug conjugates (ADCs) are well-developed delivery systems in which antibodies are combined with small molecules for highly targeted drug delivery [25]. However, because available antibodies and mature preparation technology are lacking, ADCs have been used only for cancer treatment [26, 27]. In addition, ADCs do not change the metabolic properties of the original drugs; therefore, toxicity-inducing metabolites are still formed, exposing the systemic compartment to their toxic effects [25]. Because of these problems, improving therapeutic index through the design of long-acting drugs with ideal metabolic properties, organ-specific distribution, and low systemic exposure is a great challenge, and new drug-design strategies are needed to address these issues.

Such a novel drug-design strategy would be particularly helpful for acid-related diseases (ARDs), which are typically localized to organs and characterized by stomach lesions [28, 29]. Due to the unique acidic niches of the stomach, it is very difficult to design an effective SD for the treatment of ARDs, and no antibodies to deliver drugs to the stomach have been discovered thus far. Most drugs currently used to treat ARDs, such as proton pump inhibitors (PPIs), reach high levels in the systemic compartment [30–32], inducing drug-related side effects, such as liver toxicity; thus, the antacid effects of these drugs are compromised due to these side effects. Potassium-competitive acid blockers (P-CABs), represented by vonoprazan, are distributed specifically to the stomach; their discovery was a great breakthrough in ARD treatment because they provide better symptom control, rapid onset of action, and lasting medicinal effects without nocturnal gastric acid breakthrough (NAB) [33–35]. However, structural optimization of most P-CABs is still performed under traditional methods in which drugs with a low clearance rate and high systemic exposure are pursued, and their characteristic drug distribution in tissue is not considered. Therefore, available P-CABs are associated with abnormal hepatic function, drug eruption, erythema multiforme, pancytopenia, toxic epidermal necrolysis, and, in particular, abnormal hepatic function, which is the most common side effect due to high systemic exposure to P-CABs and their highly oxidative metabolites [36]. Because of these drug-related toxicities, especially liver toxicity, the available acid-suppressive drugs, including vonoprazan, seem to not meet the clinical requirements for long-term and/or prophylactic use; these requirements are based on the highest safety standards [36]. In addition, due to the complex diets and high Helicobacter pylori infection, the incidence of stomach disease in China is as high as 85% [37]; the development of drugs for the treatment of stomach disease is of special significance in China.

**Figure 1:**
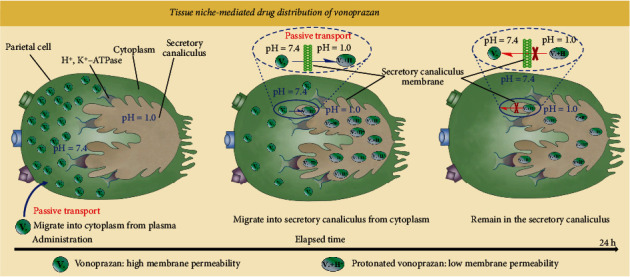
Tissue niche-mediated drug distribution (TNMDD) of vonoprazan. (1) After administration and absorption, vonoprazan migrates into the cytoplasm of parietal cells from plasma via passive transport. (2) Vonoprazan then migrates into the acidic secretory canaliculus from the cytoplasm via passive transport and becomes protonated. (3) Protonated vonoprazan, which shows low membrane permeability, cannot migrate back into the cytoplasm and thus accumulates, remaining in a protonated state over a long period even after the nonionic form in the plasma and the cytoplasm is eliminated.

Based on the need for new organ-targeted drug-design strategies, we first proposed the concept of tissue niche-mediated drug distribution (TNMDD) and developed a strategy of distribution- and metabolism-based drug design (DMBDD). As a proof of concept, we first analyzed the specific tissue-based distribution of the P-CAB vonoprazan as a representative of TNMDD (Figure 1). Vonoprazan is a lipophilic weak base with a pKa of 9.1-9.3 and log *D*_7.4_ of 0.4 [35, 38, 39], in the neutral blood environment (pH 7.4); in its nonionic form, vonoprazan exhibits high membrane permeability, whereas its ionic form exhibits a clear decline in membrane permeability in the acidic environment of the secretory canaliculi (pH 1.0) of the stomach [35]. Therefore, vonoprazan is rapidly absorbed from the blood into acidic secretory canaliculi through passive transport and then protonated; thus, protonated vonoprazan accumulates and remains in its ionic state over a long period, even after the nonionic form of vonoprazan in the plasma is eliminated [35, 40]. Based on this characteristic tissue niche-mediated drug distribution (TNMDD) of vonoprazan, we then present an example of the development of new P-CABs derived from vonoprazan using the DMBDD strategy, and this strategy ultimately led to the development of SH-337, which exhibits best-in-class potential. Our proof-of-concept experiments demonstrated the following. (1) Physicochemical property-driven (pKa and log *D*_7.4_) optimization via DMBDD supported development of a new P-CAB (SH-337) with enhanced absorption and an increased stomach-specific distribution. (2) Rapid metabolization of systemic compartment-accumulating drugs into more secure metabolites, but not those targeted to the stomach, was achieved, limiting systemic exposure to toxic metabolites. (3) These toxic metabolites were redirected into more secure metabolites *via* a predictable metabolic pathway to limit their systemic exposure. As a result, the therapeutic window was increased, and the safety profile of SH-337 was improved over that of vonoprazan. Thus, this work demonstrates that DMBDD is a promising approach for the discovery of safe, long-acting, organ-specific drugs.

## 2. Results

### 2.1. Metabolite Analysis-Based Lead Compound Identification

**Figure 2:**
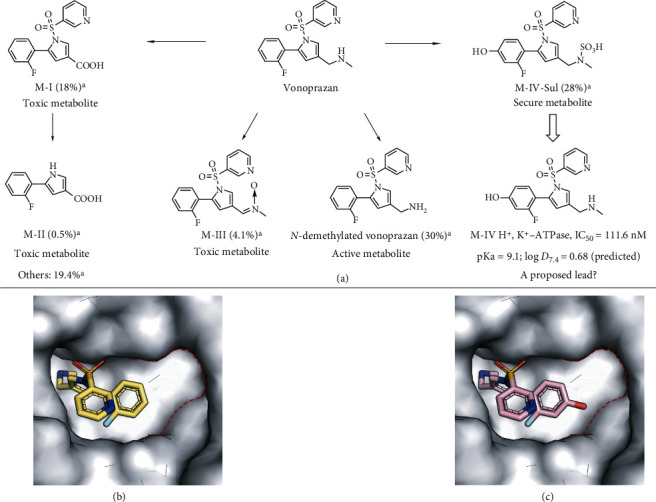
Metabolite analysis and binding model of vonoprazan and M-IV. (a) Metabolites of vonoprazan in human hepatocytes. (b) Crystal structure of gastric H^+^, K^+^-ATPase in complex with vonoprazan [41]. (c) Binding model showing M-IV bound to H^+^, K^+^-ATPase. ^a^Values obtained from an *in vitro* metabolic profile of vonoprazan with human hepatocytes [42].

Despite the TNMDD of vonoprazan in the stomach, the toxic metabolites of vonoprazan are extensively distributed in the liver, which is the location of the metabolism of most medicines, and this distribution will then lead to high and long-term systemic exposure to toxic metabolites, particularly for the liver. As a result, abnormal hematological indicators were observed in a preclinical experiment [43]. The abnormal hematological indicators are important indices for evaluating the damage degrees of the systemic side effects induced by vonoprazan, particularly hepatotoxicity. Vonoprazan is broken down into five major active metabolites: the active metabolite *N*-demethylated vonoprazan, M-I, M-II, M-III, and M-IV-Sul (Figure 2(a)) [42]. Among these metabolites, M-I is the most toxic metabolite, is present at very high levels in the plasma, liver, and kidney, and is found at 4.3- to 58-fold higher levels compared with those of vonoprazan in a rat model [44]. High exposure to M-I may be a major cause of the abnormal blood biochemical indexes observed in preclinical research and of the systemic side effects observed after the clinical application of vonoprazan [43].

Remarkably, M-IV-Sul is the most secure metabolite and is the only metabolite that is less toxic than the parent compound, vonoprazan (Figure 2(a)) [42]. Thus, M-IV, which could be rapidly metabolized to the safe phase II metabolite M-IV-Sul or M-IV-Gluc via sulfation or glucuronidation, captured our attention. M-IV retained the privileged scaffold of vonoprazan and was predicted to exhibit the same pKa value (9.1, predicted; Figure 2(a)) as vonoprazan (pKa = 9.1 ~ 9.3), which suggested that M-IV may have potential pharmacological activity and that it may exhibit the same TNMDD as vonoprazan in the stomach. We proposed that M-IV could serve as a lead compound for the development of new P-CABs with better stomach distribution and nontoxic metabolites (M-IV-Sul or M-IV-Gluc) by identifying new substituents. Therefore, we evaluated the *in vitro* inhibitory effect of M-IV. The results showed that M-IV displayed good inhibitory activity against H^+^, K^+^-ATPase (half-maximal inhibitory concentration [IC_50_] = 111.6 nM; Figure 2(a)). More importantly, a model of M-IV docked with H^+^, K^+^-ATPase obtained by molecular docking experiments revealed the hydroxyl group of M-IV bound in a subpocket of H^+^, K^+^-ATPase (Figure 2(c)), which afforded an ideal position for the installation of suitable substituents that could insert into the subpocket (Figures 2(b) and 2(c)). Hence, our subsequent optimization focused on the identification of new substituents to occupy this subpocket.

### 2.2. Distribution- and Metabolism-Based Lead Compound Optimization

**Figure 3:**
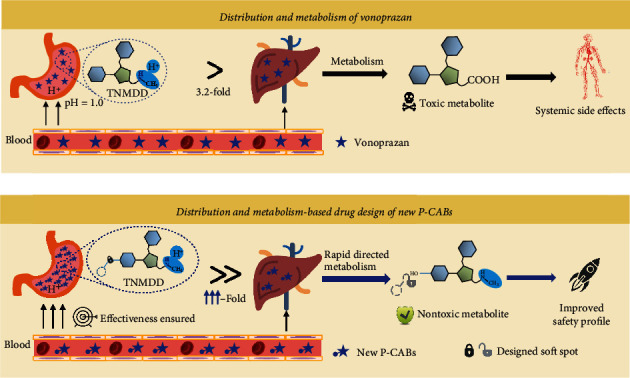
Overall strategy for distribution- and metabolism-based drug design of new P-CABs. Due to the acidic niches of the stomach, vonoprazan is specifically targeted to the stomach, resulting in a 3.2-fold greater distribution in the stomach than in the liver; however, vonoprazan metabolism produces toxic metabolites, and long-term systemic exposure to high levels of these toxic metabolites can result in systemic side effects. Based on our newly developed design strategy, we introduced an additional substituent to simultaneously promote absorption and improve the distribution ratio to ensure effectiveness. This substituent also introduces a metabolic soft spot for direct and rapid metabolization of the drug that accumulates in the systemic compartment, but not that in the stomach, into nontoxic metabolites to improve the safety profile of the new P-CAB.

The capacity of a drug to cross a biological membrane is usually determined by its lipophilicity. A distribution coefficient (log *D*_7.4_) between 1 and 3 is optimal for gastrointestinal absorption [45]. Therefore, a key step in structural optimization is modulation of the physicochemical properties to optimize log *D*_7.4_, thereby accelerating absorption, and to maintain the pKa, thus increasing stomach-specific distribution. When a metabolic soft spot is introduced in the hydroxyl group of M-IV, the systemically exposed molecule will undergo fast hepatic metabolism to produce M-IV, which will be further metabolized into secure metabolites via phase II metabolism. By contrast, protonated molecules in secretory canaliculi cannot be cleared due to the absence of related metabolic enzymes (Figure 3). As a result, systemic exposure to toxic metabolites will be significantly reduced, and the therapeutic window will be increased accordingly.

**Figure 4:**
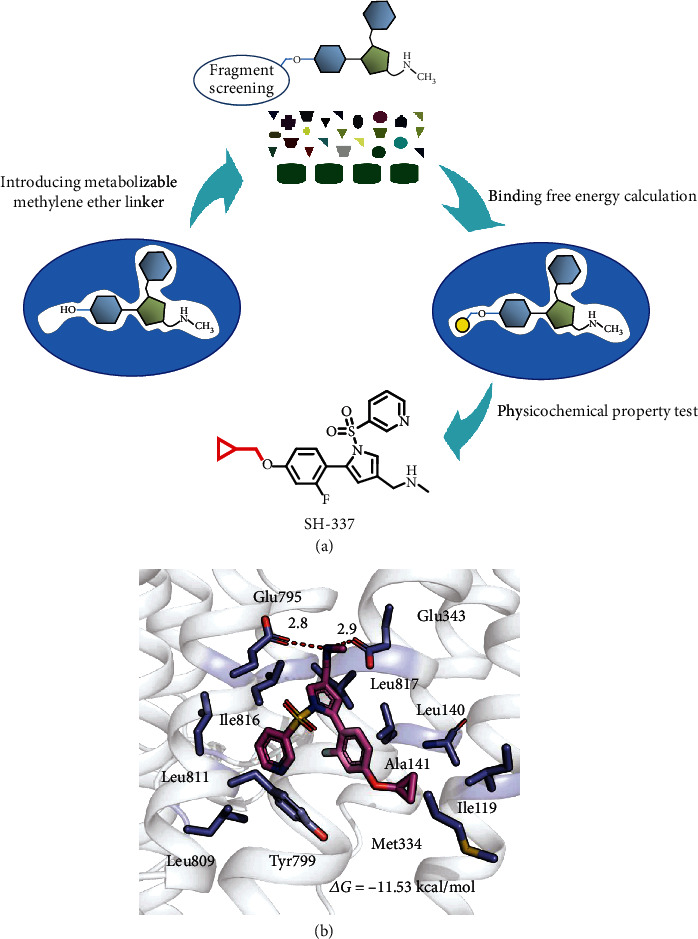
Molecular design of new P-CABs. (a) Molecular design of new P-CAB SH-337 based on DMBDD strategy. (b) Binding model showing the designed P-CAB SH-337 with H^+^, K^+^-ATPase.

Based on the above analysis, methylene, which was found to easily undergo *O*-dealkylation by P450, was employed as a linker to introduce a metabolic soft spot in the hydroxyl group, and fragment virtual screening was performed using the ACFIS [46] and PADFrag [47] databases to engage in additional hydrophobic or electrostatic interactions with the key residues in the subpocket to maintain H^+^, K^+^-ATPase inhibitory activity (Figure 4(a), Supplementary Table S1). In addition, the pKa and log *D*_7.4_ were optimized as important indicators of drug absorption and distribution. More specifically, the binding affinity with H^+^, K^+^-ATPase and physicochemical properties (pKa and log *D*_7.4_) were tested to identify candidates with optimized gastrointestinal absorption and distribution as well as inhibitory effects (Supplementary Table S1).

Through these efforts and robust chemical derivatization and characterization, we finally identified SH-337, which displayed 2.0-fold greater enzyme inhibitory activity than vonoprazan (33.8 nM *vs.* 65.5 nM, respectively; Table 1) and exhibited excellent Na^+^, K^+^-ATPase selectivity (925.6-fold; Table 1). More importantly, SH-337 exhibited a log *D*_7.4_ value of 1.3 within the range of 1 to 3, which is optimal for gastrointestinal absorption, and a high pKa value of 9.0 (Table 2), which indicates that it was rapidly absorbed and highly enriched in the stomach (6694.9 ng/mL; Table 2). In addition, its metabolic stability was substantially decreased in rat and human microsomes (*t*_1/2_ = 3.1 and 81.4 min *vs.* 54.5 and 339.1 min, SH-337 *vs.* vonoprazan, giving 17.6- and 4.2-fold reduction, respectively; Table 2). Decreased metabolic stability resulting in a really low plasma distribution (7.1 ng/mL 1 h after the oral administration of 4.0 mg/kg *vs.* 11 ng/mL 1 h after the oral administration of 2.0 mg/kg, SH-337 *vs.* vonoprazan, respectively; Table 2), the stomach-plasma distribution ratio reached 942.9 (87 for vonoprazan, resulting in a 10.0-fold increase; Table 2). This finding demonstrated that the use of a methylene ether linker led to formation of a metabolic soft spot, and our directional metabolic strategy indeed limited systemic drug exposure. Docking experiments revealed that SH-337 fit well into the subpocket of H^+^, K^+^-ATPase (Figure 4(b)). Ultimately, pharmacodynamics analysis indicated that SH-337 displayed excellent *in vivo* acid suppression activity; thus, further evaluation of SH-337 was the subject of subsequent experiments.

### 2.3. SH-337 Is Successfully Directed to Be Primarily Metabolized into M-IV and M-IV-Gluc

**Figure 5:**
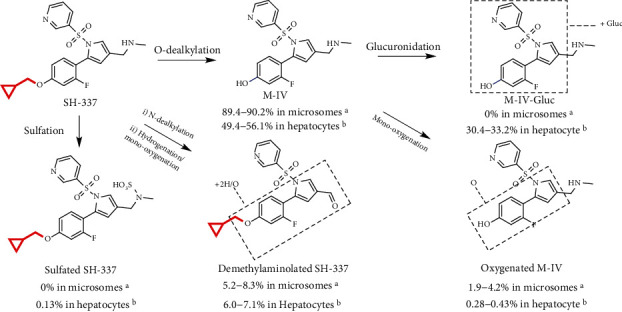
Proposed SH-337 metabolic pathway in the hepatocytes or liver microsomes of rat and human. ^a^Percentages of all SH-337 metabolites in rat and human microsomes after a 30 and 60 min incubation period. ^b^Percentages of all SH-337 metabolites in rat and human hepatocytes after a 60 and 120 min incubation period.

*In vitro* metabolic pathways in the hepatocytes or liver microsomes are often used to predict metabolic behavior *in vivo* (Figure 5). The *in vitro* metabolite profiling of SH-337 showed that the *O*-dealkylated metabolite M-IV was the major metabolite, accounting for 89.4-90.2% of all metabolites observed in liver microsomes from two species (Table 3; Figure 5; Supplementary Table S2); overall, the metabolic profiles of the two tested species were qualitatively similar. More importantly, only small amounts of oxygenated and demethylaminolated derivatives, which may be the major cause of systemic side effects, were observed (Table 3; Figure 5); in contrast, these metabolites were abundant after vonoprazan was metabolized. Furthermore, M-IV-Gluc, formed from the glucuronidation of M-IV, was identified as the major phase II metabolite in both rat and human hepatocytes. M-IV and M-IV-Gluc (M-IV+M-IV-Gluc) accounted for 82.6-86.5% of all the metabolites observed in rat and human hepatocytes (Table 3; Supplementary Table S3), indicating that SH-337 can be rapidly metabolized into M-IV via the designed metabolic soft spot and then subsequently metabolized into the secure phase II metabolite M-IV-Gluc. These results demonstrated that the DMBDD strategy effectively redirected undesired metabolism, correspondingly reduced the systemic exposure to toxic metabolites, and would accordingly increase the therapeutic window.

### 2.4. Distribution of SH-337 and Its Metabolites in Rat Tissues

The tissue and plasma distributions of SH-337 were tested *in vivo*. Plasma, liver, and stomach samples were collected at multiple time points following oral dosing of 4.0 mg/kg SH-337 to rats for compound quantitation by LC-MS/MS. After oral dosing, the SH-337 concentration peaked (9823.0 ng/mL, Table 4) in the stomach after 15 min, indicating that SH-337 overcame the first-pass effect and rapidly accumulated in stomach tissue. Specifically, the concentrations of SH-337 0.25, 1, 2, 4, and 24 h after administration were 5244.0, 846.0, 262.0, 145.0, and 1.3 ng/mL in the liver, respectively, and 9823.0, 3345.0, 3032.0, 2807.0, and 136.4 ng/mL in the stomach, respectively (Table 4). By contrast, a very low SH-337 concentration was observed in plasma throughout the experimental observation period (a maximum of 15.5 ng/mL 1 h after administration, Table 4). Thus, SH-337 was rapidly cleared from the liver but remained in the stomach after 24 h. Due to the clearance of SH-337 from the liver, the stomach-liver distribution ratio (*C*_Stomach_/*C*_Liver_) reached 104.9 after 24 h, which suggested that SH-337 could be accumulated and retained in the stomach for more than 24 h even after termination of the exposure. In other words, SH-337 has great potential to prevent nocturnal gastric acid breakthrough (NAB) with low toxicity.

Table 5 summarizes the metabolite profiles of SH-337 and vonoprazan in tissues after a single oral dose was administered to rats. Exposure to SH-337 (the final area under the curve (AUC-last)) was dramatically higher in the stomach tissue than in the liver tissue or plasma; the stomach-plasma and stomach-liver distribution ratios were 1357.1 and 7.1, respectively, while those for vonoprazan had been previously reported to be 165.0 and 3.2, respectively (Table 5). Notably, the major metabolite of SH-337, M-IV, was rapidly cleared, and not much of M-IV accumulated in the liver (AUC_0–24_ = 3411.2 ng/mL after oral dosing of 4.0 mg/kg SH-337; Table 5), in contrast to the major metabolite of vonoprazan, M-I (AUC_0–24_ = 18404.0 ng/mL after oral dosing of 2.0 mg/kg vonoprazan; Table 5). Accordingly, the exposure to SH-337+M-IV (the total amount of SH-337 and its major metabolite M-IV) in the liver was much lower than that of vonoprazan+M-I (Table 5), which may have caused a profound reduction in liver toxicity. More importantly, the really low exposure to M-IV in the stomach tissue and the impaired H^+^, K^+^-ATPase activity of M-IV demonstrated that SH-337 worked in the form of the prototype drug rather than prodrug. In summary, SH-337 was rapidly absorbed and retained in the stomach for more than 24 h but rapidly cleared from the systemic compartment. This outcome shows that the DMBDD strategy redirects undesired metabolism and correspondingly reduces systemic exposure. Thus, this strategy is very promising for increasing the therapeutic window and improving the safety profile.

### 2.5. Inhibitory Effect of SH-337 on Gastric Acid Secretion *In Vivo*

After demonstrating that SH-337 exhibits good distribution and metabolic properties, we evaluated the pharmacological efficacy of SH-337 *in vivo* by measuring its inhibitory effect on gastric acid secretion in a histamine- or 2-DG-induced gastric acid secretion rat model. In the histamine-induced gastric acid secretion rat model, SH-337 inhibited acid secretion in a dose-dependent manner (Table 6). After oral administration of one dose at 2 mg/kg, SH-337 showed comparable potency (80%; Table 6) to that of vonoprazan (77%; Table 6), and at doses of 4 mg/kg, SH-337 showed almost complete inhibition (93%; Table 6). In the 2-DG-induced gastric acid secretion rat model, oral administration of SH-337 also led to a significant dose effect. After one dose at 2 mg/kg, SH-337 displayed comparable potency to that of vonoprazan (78% *vs.* 80%; Table 6); after one dose at 4 mg/kg, SH-337 showed almost complete gastric acid secretion inhibition (91%; Table 6). These results indicated that the pharmacological effect of SH-337 was comparable to that of vonoprazan.

### 2.6. Toxicity Evaluation of SH-337

To assess the potential clinical application of SH-337, a detailed safety evaluation of SH-337 was then performed. The single-dose toxicity of SH-337 was first evaluated in Sprague–Dawley (SD) rats. A single dose of 1000 mg/kg SH-337 was well tolerated, and no animal death was observed. Furthermore, the repeated-dose toxicity of the daily oral administration of 100 mg/kg and 200 mg/kg SH-337 was evaluated for 2 weeks. In the 100 mg/kg group, no evidence of SH-337 toxicity was observed, as no abnormalities in gross morphology, body weight, food consumption, heart rate, and hematological indicators were observed. Therefore, the no observed adverse effect level (NOAEL) was initially determined to be at least 100 mg/kg, a 3.3-fold higher level than that of 30 mg/kg vonoprazan [43]. During the whole treatment period (2 weeks), hematological indicators remained normal in both of the dosing groups (100 mg/kg and 200 mg/kg) while high ALT and total cholesterol were observed in males in the 100 mg/kg dose of vonoprazan [43]. Overall, SH-337 exhibited a much better safety profile than vonoprazan in treated rats. These results suggested that SH-337 could exhibit lower toxicity in potential clinical application.

## 3. Discussion

In the context of precision medicine, the development of drugs that target specific organs is particularly important for the development of high-efficiency and low-toxicity drugs. Conventional pharmacochemical strategies often lead to the pursuit of drugs with a low clearance rate and high systemic exposure, and high systemic exposure to administered drugs and their metabolites often results in unrelated and undesired activity at sites beyond the target and induce toxicity. Undertaking predictable and rational drug design for targeting specific organs and constructing drugs to achieve an organ-specific distribution remain challenging, and many improvements are necessary [48]. Currently, organ-specific drug distribution can be achieved only through topical administration or indirect drug delivery strategies [48]. Therefore, we present a proof-of-concept example of the use of TNMDD to develop a new P-CAB. After obtaining a compound that exhibited preferential targeting of the stomach tissue, we utilized the DMBDD strategy to discover a highly safe, organ-specific drug that induced little toxicity *in vivo*.

Through physicochemical property-driven (pKa and log *D*_7.4_) distribution optimization combined with the introduction of a metabolic soft spot, the compound SH-337 was developed, which rapidly accumulated and was retained in the stomach for more than 24 h but was rapidly cleared from the systemic compartment. The stomach-plasma and stomach-liver distribution ratios of SH-337 reached 1357.1 and 7.1, respectively, while those of vonoprazan were previously reported to be 165 and 3.2, respectively. More importantly, the introduction of a metabolic soft spot guided fast hepatic metabolism of systemic molecules into M-IV and inhibited the production of the toxic metabolite M-I analog and related metabolites. In toxicity studies, the NOAEL of SH-337 was 3.3-fold greater than that of vonoprazan (100 mg/kg *vs.* 30 mg/kg, respectively). In an *in vivo* study, SH-337 exhibited an excellent acid-inhibition effect and a significant dose–effect relationship. These encouraging results suggest that SH-337 is a promising candidate P-CAB with best-in-class potential.

In summary, we provide a proof-of-concept demonstration of DMBDD strategy which propose a new vision and approach for the discovery of organ-specific drugs, particularly for gastric drugs. Of course, when applied to other organs, the premise of our strategy is that an in-depth understanding of the tissue niche of the target organ is needed, and “directing and positioning group” that does not impair the activity is also essential. For example, due to the strong cation-anion interactions between the dipalmitoylphosphatidylcholine (DPPC), the chief pulmonary surfactant located in the alveolar fluid-air interface, and polyamines, polyamines can be specifically distributed in the lung, and thus, it could serve as directing and positioning groups for lung-specific drugs [48, 49]. We believe, with the in-depth study of tissue niches in different organs, such as the eye, bone, intestines, nasal mucosa, and lung, our DMBDD strategy would promote the development of highly efficient, organ-specific drugs with low toxicity. Moreover, the preclinical development of SH-337 is underway; a new therapeutic option for patients with ARDs is expected.

## 4. Methods

### 4.1. Chemical Synthesis of M-IV and SH-337

Description of chemical synthesis of M-IV and SH-337 inhibitors is provided in the supplementary information (available here).

### 4.2. *In Vitro* Enzymatic Activity Assay

The *in vitro* enzymatic activity was calculated by measuring the inorganic phosphate produced by the hydrolysis of ATP using rabbit gastric H^+^, K^+^-ATPase. 0.25 *μ*g enzyme was preincubated at 37°C for 30 min in Tris-HCl buffer (2.0 mL, 50 mM, pH 6.5) containing 5 mM MgCl_2_, 10 *μ*M valinomycin, 0 or 20 mM KCl, and different concentrations of SH-337 and vonoprazan. The reaction was initiated by adding 5 *μ*L of 5 mM ATP 2Na, and the preparation was incubated for 30 min at 37°C and halted by the addition of 100 *μ*L of malachite green reagent (0.12% malachite green, 7.5% ammonium molybdate, and 11% tween 20 were mixed in a volume ratio of 100 : 25 : 2). H^+^, K^+^-ATPase activity was calculated after subtracting the enzyme activity in the absence of KCl.

### 4.3. Human and Rat Microsomal Stability Studies

The *in vitro* metabolic stabilities of selected compounds were performed using human and rat liver microsomes in triplicate as described previously [50].

### 4.4. Molecular Docking

The X-ray structure of H^+^, K^+^-ATPase (PDB 5YLU) [41] was obtained from the Protein Data Bank (PDB, http://www.pdb.org). The original ligand in the protein was used as the reference to define the active site. The hydrogens of the receptor were added by using Discovery Studio 4.0. The GOLD 3.0 [51] was used to dock each small molecule into the active center.

### 4.5. Metabolite Identification and Profiling in Rat and Human Liver Microsomes

Liver microsomes (1 mg protein/mL) were suspended in phosphate buffer (50 mM, pH 7.4) containing MgCl_2_ (6 mM). Substrate was dissolved in DMSO and was added to a final concentration of 10 *μ*M, such that the concentration of organic solvent in the incubation mixture did not exceed 0.2%. The reactions were initiated at 37°C by the addition of 1 mM NADPH and terminated at 30 min or 60 min (30 min for rat; 60 min for human). The samples were precipitated with acetonitrile at 1 : 2 and further centrifuged; the supernatants were dried with a stream of N_2_ gas. The residues were reconstituted with 300 *μ*L of 10% acetonitrile/H_2_O (0.1% TFA) and analyzed by LC-MS/MS. The assays were pursued under the conditions provided in Supplementary Tables S4, S6, S7, and S8.

### 4.6. Metabolite Identification and Profiling in Rat and Human Hepatocytes

SH-337 was incubated with rat or human hepatocytes at a concentration of 1.0 × 10^6^ cells/mL for 60 min or 120 min (rat 60 min, human 120 min) at 37°C in a CO_2_ incubator. The samples were precipitated with acetonitrile at 1 : 2 and further centrifuged; the supernatants were dried with a stream of N_2_ gas. The residues were reconstituted with 300 *μ*L of 10% acetonitrile/H_2_O (0.1% TFA) and analyzed by LC-MS/MS. The assays were pursued under the conditions provided in Supplementary Tables S5–S8.

### 4.7. Tissue Distribution Identification in Rats

15 SPF male SD rats were randomly divided into 5 groups, three per group. Fasting was for 4 hours. Plasma, heart, liver, and stomach were obtained 0.25, 1, 2, 4, and 24 hours after oral administration of SH-337 (0.4 mg/mL in water) at a dose of 4 mg/kg. At designated time points, rats were anesthetized with ether, and the blood samples were collected via the abdominal aorta. The blood was immediately cooled under ice-chilled conditions and centrifuged at approximately 1500 × *g* at 4°C for 10 minutes to obtain the plasma. For plasma: 20 *μ*L Std samples in duplicate, QC samples in duplicate, and rat plasma samples were mixed with 60 *μ*L ACN containing IS (200 ng/mL of tolbutamide, 50 ng/mL of propranolol, and 500 ng/mL of Dic) in EP tubes. After the mixture was vortexed for 1 min and then centrifuged for 10 min (13000 rpm, 4°C), transfer 50 *μ*L supernatant to a 96-well plate with 150 *μ*L pure water, shake for 10 min, and finally analyze by LC-MS/MS. For heart and liver: tissue samples were added with 3-folds (*w*/*v*) saline in terms of the weight of samples and then homogenated. 20 *μ*L Std samples in duplicate, QC samples in duplicate, and rat heart and liver samples were mixed with 60 *μ*L ACN containing IS (200 ng/mL of tolbutamide, 50 ng/mL of propranolol, and 500 ng/mL of Dic) in EP tubes. After the mixture was vortexed for 1 min and then centrifuged for 10 min (13000 rpm, 4°C), transfer 50 *μ*L supernatant to a 96-well plate with 150 *μ*L pure water, shake for 10 min, and finally analyze by LC-MS/MS. For stomach: tissue samples were added with 3-folds (*w*/*v*) saline in terms of the weight of samples and then homogenated. 20 *μ*L Std samples in duplicate, QC samples in duplicate, and rat stomach samples were mixed with 60 *μ*L ACN containing IS (200 ng/mL of tolbutamide, 50 ng/mL of propranolol, and 500 ng/mL of Dic) in EP tubes. After the mixture was vortexed for 1 min and then centrifuged for 10 min (13000 rpm, 4°C), transfer 50 *μ*L supernatant to a 96-well plate with 150 *μ*L pure water, shake for 10 min, and finally analyze by LC-MS/MS.

### 4.8. *In Vivo* Inhibition of Histamine-Stimulated Acid Secretion in Anesthetized Rats

60 SPF SD rats weighing 180-220 g were randomly divided into 6 groups: sham, model, SH-337 (2 mg/kg and 4 mg/kg), vonoprazan (2 mg/kg), males and females are equally divided. Fasting for 24 hours, model and sham groups were treated with deionized water. Drugs and vehicle were given through intragastric administration to rats before anesthesia. 1 h after administration, pylorus was ligated. 5 min later, histamine 2HCl (30 mg/10 mL/kg) was injected subcutaneously. At 3 h after histamine administration, the rats were killed by CO_2_ asphyxiation. The gastric contents were collected in a 10 mL centrifuge tube after removing their stomachs. Centrifuge at 3000 *g* for 10 min, and then, determine the total acid output during a 3 h period by 0.1 M NaOH [52, 53]. %Inhibition = (average of model_acid_–drug_acid_)/average of model_acid_ × 100%.

### 4.9. *In Vivo* Inhibition of 2DG-Stimulated Acid Secretion in Anesthetized Rats

The 2DG-stimulated acid secretion in anesthetized rats was measured as described as for histamine-stimulated acid secretion, with the exception that 5 min after pylorus ligation, 2-DG (200 mg/10 mL/kg) was injected subcutaneously [52, 54].

### 4.10. Toxicology Evaluation

For acute toxicity test: 12 SPF SD rats were randomly divided into three groups and received a single dose of SH-337 (1000 mg/kg, i.g.), and the animal tolerance was recorded within 2 hours. For two-week repeat-dose toxicity test: 20 SPF SD rats were randomly divided into two groups and received SH-337 (100, 200 mg/kg, i.g.) once daily for 14 days. SH-337 was dissolved in 0.5% CMC-Na. The body weight of the rats was recorded every 7 days. During the experiment, clinical parameters included apparent changes in the mental state, skin or fur, behavior, genitals, mucous membranes, respiration, stool consistency, glandular organ secretions, mortality, or other toxic results that were recorded. In addition, animal bodyweight and food intake were assessed once weekly over the study period. At the end of the experiment, the rats were sacrificed, and the serum hematological parameters and serum biochemistry parameters were recorded.

## Figures and Tables

**Table 1:** *In vitro* H^+^, K^+^-ATPase and Na^+^, K^+^-ATPase activity of vonoprazan and SH-337.

Compd.	*In vitro* H^+^, K^+^-ATPase (IC_50_, nM)	*In vitro* Na^+^, K^+^-ATPase (IC_50_, nM)	Fold selectivity
Vonoprazan	65.5	47123.0	719.4
SH-337	33.8	31286.0	925.6

**Table 2:** log *D*, pKa, *in vitro* liver microsome metabolic stability, drug distribution, and *in vivo* inhibitory efficacy on histamine-stimulated gastric acid secretion in anaesthetized rats of selected compounds.

Compd.	log *D*_7.4_	pKa	Metabolic stability (*t*_1/2_, min) [rat/human]	Drug concentration (ng/mL)^b^		*C*_Stomach_/*C*_Plasma_^d^	*In vivo* efficacy (% inhibition)*^f^*
				Plasma	Stomach		
Vonoprazan	0.4^a^	9.1/9.3^a^	54.5/339.1	11.0^c^	957.0^c^	87.0^e^	78%
SH-337	1.3	9.0	3.1/84.1	7.1	6694.9	942.9	83%

^a^Cited from previous studies [35, 38, 39]. ^b^Drug concentration in the plasma and stomach of rats 1 h after oral administration of 4.0 mg/kg drug. ^c^Drug concentration in the plasma and stomach of rats 1 h after oral administration of 2.0 mg/kg as previously reported [40]. ^d^Ratio of stomach distribution to plasma distribution 1 h after oral administration of 4.0 mg/kg. ^e^Calculation is based on data obtained from a previous study [40]. ^f^Histamine-induced acid secretion in rats (2 mg/kg, intragastric administration (i.g.)).

**Table 3:** SH-337 and SH-337 metabolites in rat and human liver microsomes (RLMs and HLMs) after a 30 and 60 min incubation period and in rat and human hepatocytes (RHeps and Hheps) after a 60 and 120 min incubation period.

Compound/metabolite	Relative peak area (%)			
	RLMs	HLMs	RHeps	Hheps
SH-337	45.7	68.9	28.2	76.5
M-IV	49 (90.2^a^)	27.8 (89.4^a^)	40.3 (56.1^a^)	11.6 (49.4^a^)
Oxygenated M-IV	2.3	0.6	0.2	0.1
Demethylaminated SH-337	2.8	2.6	5.1	1.4
M-IV-Gluc	—	—	21.8 (30.4^a^)	7.8 (33.2^a^)
Sulfated SH-337	—	—	0.1	ND
Other metabolites	0.2	0.1	4.3	2.6
Total	100	100	100	100

^a^Percentage of all the metabolites except SH-337.

**Table 4:** Drug concentration and exposure in tissues after single oral administration of SH-337 to rats at 4 mg/kg.

	Concentration (ng/mL)					AUC-last (h^∗^ng/g)
Tissue	15 min	1 h	2 h	4 h	24 h	
Plasma	9.4	15.5	5.1	6.7	BLQ	32.6
Liver	5244.0	846.0	262.0	145.0	1.3	6155.6
Stomach	9823.0	3345.0	3032.0	2807.0	136.4	44240.4
C_Stomach_/C_Liver_^a^	1.9	3.95	11.6	19.4	104.9	7.1

^a^ Stomach-liver distribution ratio. BLQ: below limit of quantification.

**Table 5:** Metabolite profiles in tissues after *i.g.* administration of 2 mg/kg vonoprazan or 4 mg/kg SH-337 to rats.

Compound	Dose	Metabolite	AUC_0–24_ (ng equiv. h/mL or g)			*C*_Stomach_/*C*_Plasma_^b^	*C*_Stomach_/*C*_Liver_^c^
			Plasma	Liver	Stomach		
Vonoprazan^a^	2.0	Vonoprazan	79.0	4039.0	13085.0	165.0	3.2
		M-I	4392.0	18404.0	918.0	—	—
		Vonoprazan+M-I	4471.0	22713.0	14003.0	3.1	0.6

SH-337	4.0	SH-337	32.6	6155.6	44240.4	1357.1	7.1
		M-IV	20.3	3411.2	94.1	—	—
		SH-337+M-IV	52.9	10857.8	44334.5	838.1	4.1

^a^Cited from a previous study [43]. ^b^Stomach-plasma distribution ratio. ^c^Stomach-liver distribution ratio.

**Table 6:** Inhibitory effect of compounds on histamine- and 2-DG-stimulated gastric acid secretion in rats.

Group	Dose (i.g., mg/kg)	Histamine-stimulated model		2-DG-stimulated model	
		Total acid output (10^−4^ mol)	Inhibition (%)	Total acid output (10^−4^ mol)	Inhibition (%)
Vehicle	—	1.96 ± 0.74	—	3.00 ± 0.96	—
Vonoprazan	2	0.29 ± 0.43^∗∗^	77	0.62 ± 0.55^∗∗^	80
SH-337	2	0.31 ± 0.39^∗∗^	80	0.65 ± 0.31^∗∗^	78
SH-337	4	0.14 ± 0.48^∗∗^	93	0.29 ± 0.29^∗∗^	91

^∗^ *p* < 0.05 and ^∗∗^*p* < 0.01.

## Data Availability

All data needed to evaluate the conclusions in the paper are present in the paper and/or the Supplementary Materials. Raw data are available from the corresponding authors on reasonable request.

## Abbreviations

TNMDD: Tissue niche-mediated drug distribution
DMBDD: Distribution- and metabolism-based drug design
P-CAB: Potassium-competitive acid blocker
PPI: Proton pump inhibitor
SD: Soft drug
ADC: Antibody–drug conjugate
ARDs: Acid-related diseases
GERD: Gastroesophageal reflux disease
PUD: Peptic ulcer disease
NAB: Nocturnal gastric acid breakthrough
AUC: Area under the curve.

## Supplementary Materials

Supplementary Materials Experimental methods; ^1^H and ^13^C NMR spectra of all new compounds; HRMS spectra of final compounds.
