# Supplementary material for: Distribution- and Metabolism-Based Drug Discovery: A Potassium-Competitive Acid Blocker as a Proof of Concept
Source: Research (Wash D C). 2022 Jul 22;2022:9852518. doi: 10.34133/2022/9852518 (PMC9343080; doi:10.34133/2022/9852518)
Supplement: Supplementary Materials — Experimental methods; 1H and 13C NMR spectra of all new compounds; HRMS spectra of final compounds. [file 9852518.f1.docx]

**Distribution- and Metabolism-Based Drug Discovery: A Potassium-Competitive Acid** **Blocker as a Proof-of-Concept**

Ming-Shu Wang^1,†^, Yi Gong^1,†^, Lin-Sheng Zhuo^1,†^, Xing-Xing Shi^1^, Yan-Guang Tian^1^, Chang-Kang Huang^2^, Wei Huang^1,^*, and Guang-Fu Yang^1,^*

**Supplementary Table S1*.*** Calculated binding free energies with H^+^, K^+^ATPase (kcal/mol), *in vitro* H^+^, K^+^ ATPase inhibition activity and physicochemical properties of new designed P-CABs.

| P-CABs | ∆E_ele_ | ∆E_vdW_ | ∆E_MM_ | ∆G_PB_ | ∆G_SA_ | ∆E_bind_ | -T∆S | ∆G_bind_ | Activity  IC_50,_(nM) | log *D*_7.4_ | pKa |
| --- | --- | --- | --- | --- | --- | --- | --- | --- | --- | --- | --- |
| S1 |  | -76.74 | -78.09 | 129.88 | -7.74 | -32.69 | 21.30 | -11.39 | 57.8 | 0.50 | 9.26 |
| S2 |  | -72.62 | -77.99 | 128.20 | -8.60 | -31.01 | 20.82 | -10.16 | 129 | 0.91 | 9.13 |
| SH-337 |  | -71.25 | -76.33 | 123.97 | -8.05 | -31.66 | 20.13 | -11.53 | 33 | 1.28 | 9.03 |
| vonoprazan |  | -64.59 | -71.80 | 114.66 | -6.51 | -28.24 | 17.21 | -11.03 | 65.5 | 0.4 | 9.1/9.3 |

Note: The binding free energy (*∆G_binding_*) was calculated by the combination of MM/PBSA method for the enthalpy and an empirical method for the entropy as following formula:

∆G*_binding_* = *∆H* - T*∆S* = *∆E_MM_ + ∆G_sol_ -* T*∆S*

*∆E_MM_ = ∆E_ele_ + ∆E_vdw_*

*∆G_sol_ = ∆G_PB_ + ∆G_SA_*

In which *∆G_binding_* was the binding free energy of ligand, *∆H* and -T*∆S* were the enthalpy and the entropy, respectively. The changes in the gas phase molecular mechanics (MM) energy *∆E_MM_* could be calculated though electrostatic energies *∆E_ele_*, and the van der Waals energies *∆E_vdw_*. While solvation free energy *∆G_sol_* is the sum of the electrostatic solvation energy *∆G_PB_* (polar contribution) and the nonpolar contribution *∆G_SA_* between the solute and the continuum solvent. The polar contribution is calculated using the PB model, while the nonpolar energy is usually estimated using the solvent-accessible surface area (SASA).

Supplementary Table S2*.* Observed Metabolites of SH-337 in Rat and Human Liver Microsomes after 30 min or 60 min Incubation.

| **Metabolite** | **Retention Time (min)** | ***m/z* (+)** | **Metabolic Pathway** | **Relative Peak Area Abundance (%)** | |
| --- | --- | --- | --- | --- | --- |
|  |  |  |  | **RLM** | **HLM** |
| M1 | 5.98 | 378.0918 | *O*-dealkylation and mono-oxygenation | 0.5 | D |
| M2 | 6.30 | 378.0918 | *O*-dealkylation and mono-oxygenation | 1.8 | 0.6 |
| M3* | 7.14 | 362.0969 | *O*-dealkylation | 49.0 | 27.8 |
| M4 | 7.87 | 432.1388 | Mono-oxygenation | 0.2 | ND |
| M5 | 9.97 | 432.1388 | Mono-oxygenation | D | 0.2 |
| M6 | 11.49 | 403.1122 | *N*-dealkylation and hydrogenation | 0.4 | 0.4 |
| P (SH-337) | 11.78 | 416.1439 | - | 45.7 | 68.9 |
| M7 | 16.15 | 403.1122 | *N*-dealkylation and hydrogenation | ND | 0.5 |
| M8 | 16.26 | 417.0915 | *N*-dealkylation and mono-oxygenation | 2.4 | 1.6 |

Note: human species was incubated for 60 min, the others were incubated for 30 min; P = parent; RLM, rat Liver Microsomes; HLM, human Liver Microsomes; D = Detected; ND = not detected; the relative abundances of the parent and metabolites were calculated based on their selected ion chromatographic peak areas. *: M3 was also detected in T0 sample and standard sample.

Supplementary Table S3. Observed Metabolites of SH-337 in Rat Hepatocytes after 60 min and Human Hepatocytes after 120 min Incubation

| **Metabolite** | **Retention Time (min)** | ***m/z* (+)** | **Metabolic Pathway** | **Relative Peak Area Abundance (%)** | |
| --- | --- | --- | --- | --- | --- |
|  |  |  |  | **RHep** | **HHep** |
| M1 | 5.33 | 538.129 | *O*-dealkylation and glucuronidation | 21.8 | 7.8 |
| M4 | 7.05 | 392.1064 | *O*-dealkylation, mono-oxygenation and methylation | 3.3 | 0.2 |
| M5* | 7.11 | 362.0969 | *O*-dealkylation | 40.3 | 11.6 |
| M6 | 7.86 | 432.1388 | Mono-oxygenation | 0.4 | ND |
| M7 | 8.91 | 376.0762 | *O*-dealkylation, mono-oxygenation and dehydrogenation | 0.2 | 0.1 |
| M8 | 9.92 | 432.1388 | Mono-oxygenation | 0.6 | 0.4 |
| M9 | 11.44 | 403.1122 | *N*-dealkylation and hydrogenation | 0.9 | 0.5 |
| P (SH-337) | 11.78 | 416.1439 | - | 28.2 | 76.5 |
| M10 | 14.54 | 496.1007 | Sulfation | 0.1 | 2.0 |
| M11 | 16.07 | 403.1122 | *N*-dealkylation and hydrogenation | 0.1 | ND |
| M12 | 16.23 | 417.0915 | *N*-dealkylation and mono-oxygenation | 4.1 | 0.9 |

Note: P = parent; RHep, rat hepatocytes; HHep, human hepatocytes; ND = not detected; the relative abundances of the parent and metabolites were calculated based on their selected ion chromatographic peak areas. M5* was also detected in T0 sample and standard sample, it was partly contributed from the impurity.

**Supplementary Table S4.** Liver Microsomes Incubation System.

| Test Compound Concentration: | 10 μM |
| --- | --- |
| Microsomal Proteins: | 1.0 mg/mL |
| Microsomal Species: | Rat and Human |
| β-NADPH Concentration: | 1.0 mM |
| Phosphate Buffer: | 50 mM, pH 7.4 |
| Incubation Temperature: | 37 °C |
| Incubation Time: | Rat (0, 30 min), Human (0, 60 min), |
| Positive Control: | 7-EC (0, 60 min) |

**Supplementary Table S5.** Hepatocytes Incubation System

| Test Compound Concentration: | 10 μM |
| --- | --- |
| Hepatocytes Concentration: | 1.0 ×10^6^ cells/mL |
| Species: | Rat and Human |
| Cell Culture: | Williams’ Medium E |
| Incubation Temperature: | 37 °C, CO_2_ Incubator |
| Incubation Time: | Rat (0, 60) min, Human (0, 120) min |
| Positive Control: | 7-Ethoxycoumarin (0, 120 min) |

**Supplementary Table S6.** HPLC Conditions for Metabolite Identification and Profiling.

| UHPLC: | Ultimate 3000 |
| --- | --- |
| Column: | ACQUITY UPLC HSS T3 1.8 μm, 2.1×100 mm |
| Column Temperature: | 25 °C |
| Flow Rate: | 0.4 mL/min |
| Injection Volume: | 10 μL |
| Run Time: | 22 min |
| Mobile Phase: | A = Water containing 0.1% formic acid |
|  | B = Acetonitrile containing 0.1% formic acid |

**Supplementary Table S7.** HPLC Gradient for Metabolite Identification and Profiling.

| Time (min) | A (%) | B (%) |
| --- | --- | --- |
| 1.0 | 95 | 5 |
| 6.0 | 75 | 25 |
| 14.0 | 55 | 45 |
| 16.0 | 10 | 90 |
| 18.0 | 5 | 95 |
| 20.1 | 95 | 5 |
| 22.0 | 95 | 5 |

**Supplementary Table S8.** Mass Condition for Metabolite Identification and Profiling.

| Mass spectrometer: | Q-Exactive Plus |
| --- | --- |
| Operation software system: | Xcalibur |
| Ionization mode: | ESI^+^ |
| Scan range: | 80-1000 *m/z* |
| Spectrum data type: | Profile for full scan; centroid for dd-MS^2^ scan |
| AGC target: | 5.0×10^5^ for full scan; 2.0×10^4^ for dd-MS^2^ scan |
| Sheath gas flow rate: | 40 |
| Aus gas flow rate: | 15 |
| Sweep gas flow rate: | 0 |
| Capillary temp.: | 375 °C |
| Aus gas heater temp.: | 350 °C |
| S-lens RF level: | 55 |
| Mass resolution: | 70,000 for full MS; 17,500 for dd-MS^2^ |
| Maximum IT: | 100 ms for full MS; 50 ms for dd-MS^2^ |
| Isolation window: | 1(*m/z*) |
| (N)CE (eV): | 25±10, 35±10, 45±10 |
| Error level: | 5.0 ppm |

**Supplementary Note 1. Synthesis and characterization of M-IV, S1, S2 and SH-337.**

**General Chemistry.** Unless otherwise noted, reagents and solvents used in experiments were purchased from commercial sources and used without further purification. Flash chromatography was performed using 200-400 Mesh silica gel from Qingdao Makall Group Co., Ltd.; China. Silica gel plates-based thin-layer chromatography (TLC) was used to monitor all reactions with fluorescence F254 or F365 light. the reactions involving air- or moisture-sensitive reagents were performed under a nitrogen or argon atmosphere. ^1^H NMR spectra (400 or 600 MHz) and ^13^C NMR (100 or 150 MHz) spectra were recorded on a Bruker BioSpin AG (Ultrashield Plus AV 400M or 600M) spectrometer as Methanol-*d*_4_ (CD_3_OD-*d_4_*) or deuterochloroform (CDCl_3_) or dimethyl sulfoxide-*d_6_* (DMSO-*d_6_*) solutions using tetramethylsilane (TMS) as an internal standard (*δ* = 0) unless noted otherwise. In the tabulated NMR results, s indicates singlet; br, broad singlet; d; doublet; t, triplet; q, quartet; m, multiplet; dd, doublet of doublet. High-resolution mass spectra (HR-MS) were obtained on an Agilent 6224 TOF LC/MS (USA). Yields were of purified compounds and were not optimized.

**Procedure for pKa and log D_7.4_ measurements.** The pKa was determined using pH-metric Method from DMSO Stock Solution. Procedure for Log D determinations: 15 µL of stock solutions of test compounds and control compound was placed in order into their proper 96-well rack. 500 µL of PBS saturated 1-octanol was added into each vial of the cap-less Log D plate followed by the addition of 500 µL of 1-octanol saturated PBS (pH 7.4). One stir stick was added to each vial and a molded PTFE/Silicone plug was used to seal each vial. Then the Log D plate was transferred to the Eppendorf Thermomixer Comfort plate shaker and shaken at 25°C at 1,100 RPM for 1 hour. After completion of 1 hour, plugs were removed and the stir sticks were removed using a big magnet. The samples were then centrifuged at 25°C at 20,000 g for 20 minutes to separate the phases, and pipette and syringe were used to remove the upper (1-octanol) and lower (buffer) phases to the empty tubes, respectively. Aliquots of 5 µL were taken from upper phases followed by addition of 495 µL of a mixture of H_2_O and acetonitrile (1:1 in v/v). Vortex for 1 minute, and then aliquots of 50 µL were taken from the diluent followed by addition of 450 µL of a mixture of H_2_O and acetonitrile (1:1 in v/v). And aliquots of 50 µL were taken from lower phases followed by addition of 450 µL of a mixture of H_2_O and acetonitrile (1:1 in v/v). 200 μL of diluent was transferred to a new 96-well plate for LC-MS/MS analysis. The dilution factor was changed according to the Log D value and the LC-MS signal response. The concentrations of test compound and control compound in 1-octanol/buffer solution were evaluated by LC-MS/MS. The Log D values of the test compounds and control compound were calculated as follows:

$$LogD=Log\left(\frac{AREA_{Oct}\times INJ VOL_{Buf}\times DF_{Oct}}{AREA_{Buf}\times INJ VOL_{Oct}\times DF_{Buf}}\right)$$

**Supplementary Scheme 3. Synthetic pathway of M-IV, S1, S2 and SH-337.**

Reagents: i) *N*-Bromosuccinimide, -78 °C, THF, DMF; ii) 1). CH_3_NH_2_, NaBH_4_, MeOH, 0 °C, 2). tert-butoxycarbonylanhydride, CH_3_CN, RT; iii) NaH, Pyridine-3-SulfonylChloride, THF, 0 °C; iv) Bis(pinacolato)diboron, Pd(PPh_3_)_4_, NaHCO_3_, DME/H_2_O; v) Pd(PPh_3_)_4_, NaHCO_3_, DME/H_2_O; vi) HCl in EA; vii) 1) PPh_3_, Diisopropyl azodicarboxylate, PhMe, 70 °C; 2) HCl in EA; 3) Fumaric acid, MeOH; viii) 1) PPh_3_, Diisopropyl azodicarboxylate, PhMe, 70 °C; 2) HCl in EA.

**5-bromo-1H-pyrrole-3-carbaldehyde (2)**

1H-pyrrole-3-carbaldehyde **1** (5.0 g, 52.6 mmol) was added to THF (100 mL) and the resulting clear solution was cooled to -78 °C. *N*-Bromosuccinimide (NBS, 9.4 g, 52.6 mmol) (1.8 M in *N,N*-Dimethylformamide) was added dropwise at -78 °C under Ar. The mixture was stirred at -78 °C for 2.0 h, then the mixture was slowly warmed to -10°C and stirred for 1 h. After completion of reaction, water was added to the reaction solution, followed by extraction with ethyl acetate. The extract was washed with saturated saline and then dried over anhydrous magnesium sulfate. The solvent was distilled off under reduced pressure. The obtained residue was purified by silica gel column chromatography (hexane-ethyl acetate) to obtain the title compound as white solid (4.7 g, 52.0 %). ^1^H NMR (600 MHz, DMSO-*d*_6_) δ 12.40 (s, 1 H), 9.60 (s, 1 H), 7.70 (d, *J* = 1.8 Hz, 1 H), 6.51 (d, *J* = 1.8 Hz, 1 H).

***tert*-Butyl ((5-bromo-1H-pyrrol-3-yl)methyl)(methyl)carbamate (3)**

To a solution of compound **2** (3 g, 17.2 mmol) in methanol (40 ml), a solution of 33 wt% methylamine in methanol (3.6 g, 34.4 mmol) was added, and the mixture was stirred at room temperature for 1 h. Then the solution was cooled to 0°C and sodium borohydride (0.98 g, 25.8 mmol) was added. The mixture was stirred at RT for 2 h. After completion, water (50 mL) was added and the mixture was extracted with EtOAc (3 × 200 mL). The combined organic phases were dried over Na_2_SO_4_, filtered and concentrated to dryness to give crude 1-(5-bromo-1H-pyrrol-3-yl)-*N*-methylmethanamine. This crude intermediate was dissolved in acetonitrile (30 mL), *tert*-butoxycarbonyl anhydride (4.5 g, 20.6 mmol) was added. The suspension was stirred at RT for 0.5 h. The mixture was diluted with EtOAc, washed with water (150 mL×3), brine (150 mL×3), dried over Na_2_SO_4_, filtered and concentrated to dryness. The crude product was purified by silica gel chromatography (hexanes/EtOAc) to give *tert*-butyl ((5-bromo-1H-pyrrol-3-yl)methyl)(methyl)carbamate as light yellow oil (3.8 g, 76.0 %). ^1^H NMR (600 MHz, DMSO-*d*_6_) δ 11.33 (s, 1 H), 6.69 (s, 1 H), 5.95 (d, *J* = 17.4 Hz, 1 H), 4.09 (s, 2 H), 2.68 (s, 3 H), 1.41 (s, 9 H).

***tert*-Butyl ((5-bromo-1-(pyridin-3-ylsulfonyl)-1H-pyrrol-3-yl)methyl)(methyl)carbamate (4)**

*tert*-Butyl ((5-bromo-1H-pyrrol-3-yl)methyl)(methyl)carbamate **3** (3.9 g, 13.5 mmol) was dissolved in anhydrous THF (60 mL). The solution was cooled to 0 °C and sodium hydride (NaH, 60% dispersion in mineral oil, 1.9 g, 47.2 mmol) was added at 0 °C under Ar. The resulting mixture was stirred at 0 °C for 30 min, then 15-crown-5 (8.92 g, 40.5mmol) was added at 0 °C followed by pyridine-3-sulfonyl chloride (3.58 g, 20.2 mmol)，the mixture was stirred at 0 °C for 30 min then RT for 30 min. After completion, the reaction was quenched with ice, extracted with EtOAc (3 × 200 mL). The combined organic phases were washed with water (10 mL), dried over Na2SO4, filtered and concentrated to dryness. The residue was purified by silica gel chromatography (50% to 100% EtOAc in hexanes) to yield *tert*-butyl ((5-bromo-1-(pyridin-3-ylsulfonyl)-1H-pyrrol-3-yl)methyl)(methyl)carbamate as light yellow solid (4.15 g, 71.5 %). ^1^H NMR (600 MHz, DMSO-*d_6_*) δ 9.06 (s, 1H), 8.92 (d, *J* = 4.8 Hz, 1 H), 8.29 (d, *J* = 8.4 Hz, 1 H), 7.72 – 7.67 (m, 1 H), 7.49 (s, 1 H), 6.37 (d, *J* = 18.6 Hz, 1 H), 4.09 (s, 2 H), 2.68 (s, 3 H), 1.34 (s, 9 H).

**3-fluoro-4-(4,4,5,5-tetramethyl-1,3,2-dioxaborolan-2-yl)phenol (6)**

A mixture of 4-bromo-3-fluorophenol **5** (5.0 g, 31.4 mmol), bis(pinacolato)diboron (10.0 g, 41.7 mmol), Pd(PPh_3_)_4_, (1.5 g, 1.3 mmol) and potassium acetate (5.1 g, 52.4 mmol) were added to a sealed tube. Then, degassed and charged with nitrogen. 1,4-Dioxane (40 mL) was added by syringe at room temperature. The mixture was heated to 110 °C for overnight under nitrogen atmosphere. The reaction was allowed to cool and then diluted with ethyl acetate (500 mL), wash with brine (2×100mL), dried over Na_2_SO_4_, filtered, and concentrated. The residue was purified by chromatography (hexanes/EtOAc) to yield 3-fluoro-4-(4,4,5,5-tetramethyl-1,3,2-dioxaborolan-2-yl)phenol as white solid (3.0 g, 48%).^1^H NMR (600 MHz, DMSO-*d_6_*) δ 10.30 (s, 1 H), 7.49 – 7.42 (m, 1 H), 6.61 (d, *J* = 8.4 Hz, 1 H), 6.47 (d, *J* = 11.4 Hz, 1 H), 1.25 (s, 12 H).

***tert*-Butyl ((5-(2-fluoro-4-hydroxyphenyl)-1-(pyridin-3-ylsulfonyl)-1H-pyrrol-3-yl)methyl)(methyl)carbamate (7)**

A mixture of *tert*-butyl ((5-bromo-1-(pyridin-3-ylsulfonyl)-1H-pyrrol-3-yl)methyl)(methyl)carbamate **4** (1.0 g, 2.3 mmol）), 3-fluoro-4-(4,4,5,5-tetramethyl-1,3,2-dioxaborolan-2-yl)phenol **6** (0.8 g, 3.5 mmol), Pd(PPh_3_)_4_, (269.0 mg, 0.2 mmol) and sodium bicarbonate (585.0 mg, 6.9 mmol) were added to a sealed tube. Then degassed and charged with nitrogen. 1,2-Dimethoxyethane (20 mL), H_2_O (5 mL) were added by syringe at room temperature. The mixture was heated to 100 °C for 2 h under an atmosphere of nitrogen. The reaction was allowed to cool and then diluted with ethyl acetate (400 mL), wash with brine (2×150mL), dried over Na_2_SO_4_, filtered, and concentrated. The residue was purified by chromatography (hexanes/EtOAc) to yield *tert*-butyl ((5-(2-fluoro-4-hydroxyphenyl)-1-(pyridin-3-ylsulfonyl)-1H-pyrrol-3-yl)methyl)(methyl)carbamate as white solid (860 mg, 80.7%). ^1^H NMR (600 MHz, DMSO-*d*_6_) δ10.22 (s, 1 H), 8.76 (dd, *J* = 4.8, 1.8 Hz, 1 H), 8.51 (d, *J* = 2.4 Hz, 1 H), 7.88 – 7.82 (m, 1 H), 7.68 – 7.59 (m, 1 H), 7.42 (s, 1 H), 6.93 – 6.85 (m, 1 H), 6.62 – 6.55 (m, 1 H), 6.47 (dd, *J* = 11.4, 2.4 Hz, 1 H), 6.14 (s, 1 H), 4.25 (s, 2 H), 2.82 (s, 3 H), 1.45 (s, 9 H).

**3-fluoro-4-(4-((methylamino)methyl)-1-(pyridin-3-ylsulfonyl)-1H-pyrrol-2-yl)phenol dihydrochloride (M-IV)**

*tert*-Butyl ((5-(2-fluoro-4-hydroxyphenyl)-1-(pyridin-3-ylsulfonyl)-1H-pyrrol-3-yl)methyl)(methyl)carbamate **7** (200.0 mg, 0.43 mmol) was dissolved in EtOAc (2.0 mL), then HCl in EtOAc (4 M, 2.0 mL) was added and stirred at RT for 12 h to give a white suspension. The solid was collected by filtration, washed with EtOAc and dried to yield 3-fluoro-4-(4-((methylamino)methyl)-1-(pyridin-3-ylsulfonyl)-1H-pyrrol-2-yl)phenol dihydrochloride (M-IV) as white solid (120 mg, 60.1%).^1^H NMR (600 MHz, DMSO-*d*_6_) δ 9.51 – 9.37 (m, 2H), 8.89 (d, *J* = 4.7 Hz, 1H), 8.56 (d, *J* = 2.4 Hz, 1H), 7.92 (dt, *J* = 8.3, 2.0 Hz, 1H), 7.82 (d, *J* = 1.9 Hz, 1H), 7.64 (dd, *J* = 8.2, 4.9 Hz, 1H), 6.82 (t, *J* = 8.5 Hz, 1H), 6.67 – 6.58 (m, 2H), 6.53 (d, *J* = 1.9 Hz, 1H), 3.97 (t, 2H), 2.49 (s, 3H). ^13^C NMR (101 MHz, DMSO) δ 161.35 (d, *J*_C-F_ = 245.0 Hz), 160.89 (d, *J*_C-F_ = 11.0 Hz), 155.39, 147.30, 135.37, 134.67, 133.64 (d, *J*_C-F_ = 4.0 Hz), 129.23, 125.07, 124.85, 119.34, 118.39, 111.47(d, *J*_C-F_ = 2.0 Hz), 108.54 (d, *J*_C-F_ = 16.0 Hz), 102.83 (d, *J*_C-F_ = 24.0 Hz), 43.95, 32.07. HRMS (ESI) m/z calcd for C_17_H_17_FN_3_O_3_S^+^ (M+H)^+^ 362.0969, found 362.0971.

**1-(5-(2-fluoro-4-((1-methyl-1H-pyrazol-4-yl)methoxy)phenyl)-1-(pyridin-3-ylsulfonyl)-1H-pyrrol-3-yl)-N-methylmethanamine fumarate (S1)**

A mixture of *tert*-butyl ((5-(2-fluoro-4-hydroxyphenyl)-1-(pyridin-3-ylsulfonyl)-1H-pyrrol-3-yl)methyl)(methyl)carbamate **7** (400 mg, 0.86 mmol), triphenylphosphine (454mg, 1.7 mmol), (1-methyl-1H-pyrazol-4-yl)methanol (190.6 mg, 1.7 mmol) in toluene (30 mL) was heated to 80 °C, then diisopropyl azodiformate (384 mg, 1.7 mmol) were added dropwise, and the resulted mixture was stirred at 80°C for 0.5 hour. After completion, the mixture was concentrated to dryness. The residue was purified by silica gel column chromatography (hexanes/EtOAc) to give crude *tert-butyl* ((5-(2-fluoro-4-((1-methyl-1H-pyrazol-4-yl)methoxy)phenyl)-1-(pyridin-3-ylsulfonyl)-1H-pyrrol-3-yl)methyl)(methyl)carbamate. This crude intermediate was dissolved in EtOAc (2.5 mL), HCl in EtOAc (4 M, 2.5 mL) was added and stirred at RT for 12 h to give a white suspension. Then, the mixture was concentrated to dryness, DCM (200 mL) and sat.aq. NaHCO_3_ (100 mL) were added to the residue. The biphase was stirred at RT for 4 h and separated. The organic phase was washed with sat. aq. NaHCO_3_ (2 × 50 mL), dried over Na_2_SO_4_, filtered and concentrated. The residue was purified by preparative TLC to give 1-(5-(2-fluoro-4-((1-methyl-1H-pyrazol-4-yl)methoxy)phenyl)-1-(pyridin-3-ylsulfonyl)-1H-pyrrol-3-yl)-*N*-methylmethanamine as yellow oil (230 mg, 0.59 mmol), the obtained free base was dissolved in MeOH followed by addition of the same molar amount of fumaric acid. The resulting mixture was heated to reflux for 2 hours. After cooling, the reaction solution was concentrated under reduced pressure. The obtained residue was purified recrystallization from methanol solution to yield 1-(5-(2-fluoro-4-((1-methyl-1H-pyrazol-4-yl)methoxy)phenyl)-1-(pyridin-3-ylsulfonyl)-1H-pyrrol-3-yl)-N-methylmethanamine fumarate as white solid (201 mg, 40.9%). ^1^H NMR (600 MHz, Methanol-*d*_4_) δ 8.79 (s, 1H), 8.51 (s, 1H), 7.85 (s, 1H), 7.81 – 7.66 (m, 2H), 7.64 – 7.43 (m, 2H), 6.99 (s, 1H), 6.88 – 6.55 (m, 4H), 6.38 (s, 1H), 5.05 (s, 2H), 4.08 (s, 2H), 3.90 (s, 3H), 2.70 (s, 3H). ^13^C NMR (151 MHz, dmso) δ 168.52, 161.30 (d, *J*_C-F_ = 245.0 Hz), 161.03 (d, *J* = 15.0 Hz), 155.42, 147.25, 139.46, 135.55, 135.15, 134.60 (d, *J*_C-F_ = 6.0 Hz), 133.53, 131.42, 128.81, 124.96, 124.46, 120.81, 118.51, 116.25, 110.85(d, *J* = 3.0 Hz), 110.46 (d, *J* = 9.0 Hz), 102.38 (d, *J* = 26.0 Hz), 61.73, 44.05, 38.95, 32.28. HRMS (ESI) m/z calcd for C_22_H_22_FN_5_O_3_S^+^ (M+H)^+^ 456.1500, found 456.1499.

**1-(5-(2-fluoro-4-(oxetan-3-ylmethoxy)phenyl)-1-(pyridin-3-ylsulfonyl)-1H-pyrrol-3-yl)-N-methylmethanamine dihydrochloride (S2)**

A mixture of *tert*-Butyl ((5-(2-fluoro-4-hydroxyphenyl)-1-(pyridin-3-ylsulfonyl)-1H-pyrrol-3-yl)methyl)(methyl)carbamate **7** (200 mg, 0.43 mmol), triphenylphosphine (227mg, 0.87 mmol), oxetan-3-ylmethanol (76.20 mg, 0.87mmol) in toluene (15 mL) was heated to 80 °C, then diisopropyl azodiformate (174 mg, 0.87 mmol) were added dropwise, and the resulted mixture was stirred at 80°C for 0.5 hour. After completion, the mixture was concentrated to dryness. The residue was purified by silica gel column chromatography (hexanes/EtOAc) to give crude *tert*-butyl ((5-(2-fluoro-4-(oxetan-3-ylmethoxy)phenyl)-1-(pyridin-3-ylsulfonyl)-1H-pyrrol-3-yl)methyl)(methyl)carbamate. This crude intermediate was dissolved in EtOAc (2.0 mL), HCl in EtOAc (4 M, 2.0 mL) was added and stirred at RT for 12 h to give a white suspension. The solid was collected by filtration, washed with EtOAc and dried to yield 1-(5-(2-fluoro-4-(oxetan-3-ylmethoxy)phenyl)-1-(pyridin-3-ylsulfonyl)-1H-pyrrol-3-yl)-*N*-methylmethanamine dihydrochloride as white solid (89.7 mg, 41.4%). m.p. 144.9-146.9 °C; ^1^H NMR (600 MHz, Chloroform-*d*) δ 8.69 (d, *J* = 4.8 Hz, 1 H), 8.55 (d, *J* = 2.4 Hz, 1 H), 7.69 (d, *J* = 8.4 Hz, 1 H), 7.36 (s, 1 H), 7.28 (dd, *J* = 8.4, 4.8 Hz, 1 H), 6.99 (t, *J* = 8.4 Hz, 1 H), 6.63 (dd, *J* = 8.4, 2.4 Hz, 1 H), 6.54 (dd, *J* = 11.4, 2.4 Hz, 1 H), 6.24 – 6.17 (m, 1 H), 4.88 – 4.80 (m, 2 H), 4.56 – 4.48 (m, 2 H), 4.15 (d, *J* = 6.6 Hz, 2 H), 3.61 (s, 2 H), 3.45 – 3.36 (m, 1 H), 2.41 (s, 3 H).^13^C NMR (100 MHz, DMSO) δ 162.35 (d, *J*_C-F_ = 245.0 Hz), 161.39 (d, *J* = 11.0 Hz), 155.47, 147.26, 135.37, 134.62, 133.65 (d, *J* = 3.0 Hz), 128.75, 125.13, 119.61, 118.71, 110.68 (d, *J* = 15.0 Hz), 110.65 (d, *J* = 2.0 Hz), 102.38 (d, *J* = 26.0 Hz), 66.73, 58.95, 43.98, 43.50, 32.05. HRMS (ESI) m/z calcd for C_21_H_23_FN_3_O_4_S^+^ (M+H)^+^ 432.139, found 432.1388.

**1-(5-(4-(cyclopropylmethoxy)-2-fluorophenyl)-1-(pyridin-3-ylsulfonyl)-1H-pyrrol-3-yl)-N-methylmethanamine dihydrochloride (SH-337)**

The compounds SH-337 was prepared from the key intermediate *tert*-butyl ((5-bromo-1-(pyridin-3-ylsulfonyl)-1H-pyrrol-3-yl)methyl)(methyl)carbamate **7** and cyclopropylmethanol by following a similar procedure as described for **S2**. Yield, 41.2%; white solid; m.p. 160.4-162.2 °C; ^1^H NMR (600 MHz, Methanol-*d*_4_) δ 8.91 (d, *J* = 5.0 Hz, 1H), 8.68 (s, 1H), 8.13 (d, *J* = 8.2 Hz, 1H), 7.82 (s, 1H), 7.77 (dd, *J* = 8.3, 5.1 Hz, 1H), 7.00 (t, *J* = 8.5 Hz, 1H), 6.79 – 6.72 (m, 1H), 6.70 – 6.62 (m, 1H), 6.46 – 6.38 (m, 1H), 4.10 (s, 2H), 3.88 (d, *J* = 7.0 Hz, 2H), 2.71 (s, 3H), 1.34 – 1.22 (m, 1H), 0.70 – 0.57 (m, 2H), 0.45 – 0.31 (m, 2H). ^13^C NMR (100 MHz, CD_3_OD_SPE) δ 161.75 (d, *J*_C-F_ = 246.0 Hz), 161.35 (d, *J* = 11.0 Hz), 149.39, 143.21, 139.66, 136.02, 132.62 (d, *J* = 3.0 Hz), 128.81, 125.93, 123.77, 117.76, 116.89, 109.15 (d, *J* = 3.0 Hz), 108.65 (d, *J* = 16.0 Hz), 100.74 (d, *J* = 25.0 Hz), 72.24, 43.44, 30.84, 8.81, 1.42. HRMS (ESI) m/z calcd for C_21_H_23_FN_3_O_3_S^+^ (M+H)^+^ 416.1439, found 416.1438.

**Copies of NMR Data for SH-337**

**
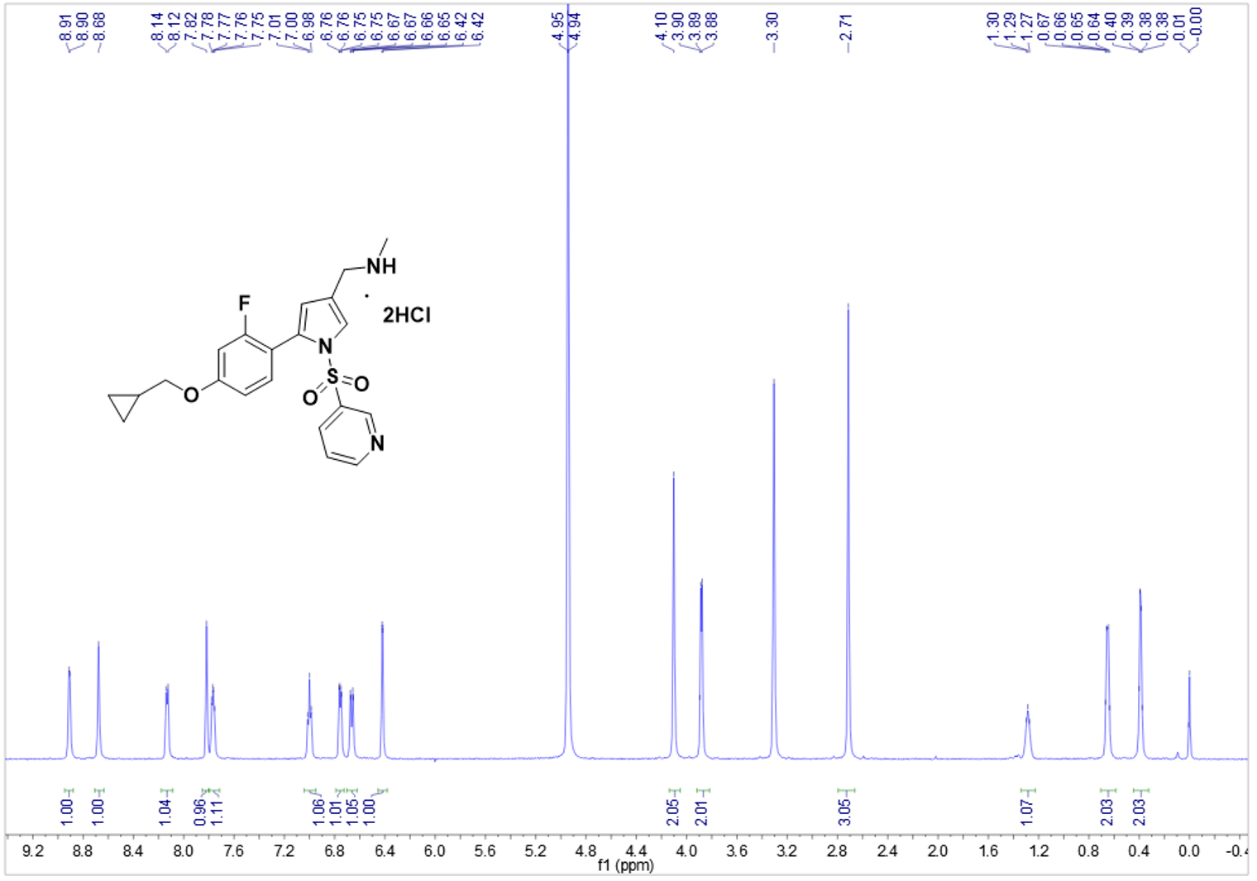
**

**Supplementary Figure 1:** 600 MHz spectrum of ^1^H-NMR of compound SH-337 (Methanol-*d*_4_)

**
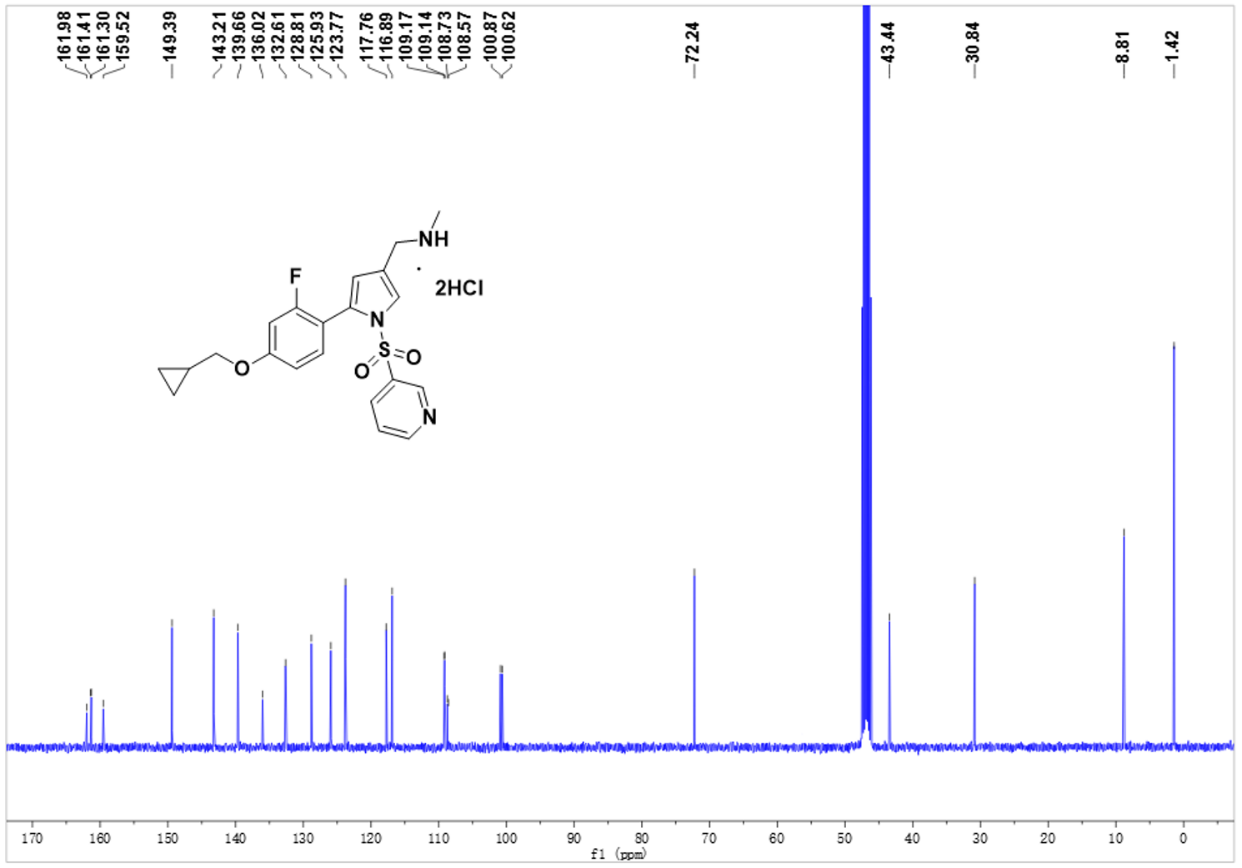
**

**Supplementary Figure 2:** 100 MHz spectrum of ^13^C-NMR of compound SH-337 (Methanol-*d*_4_)


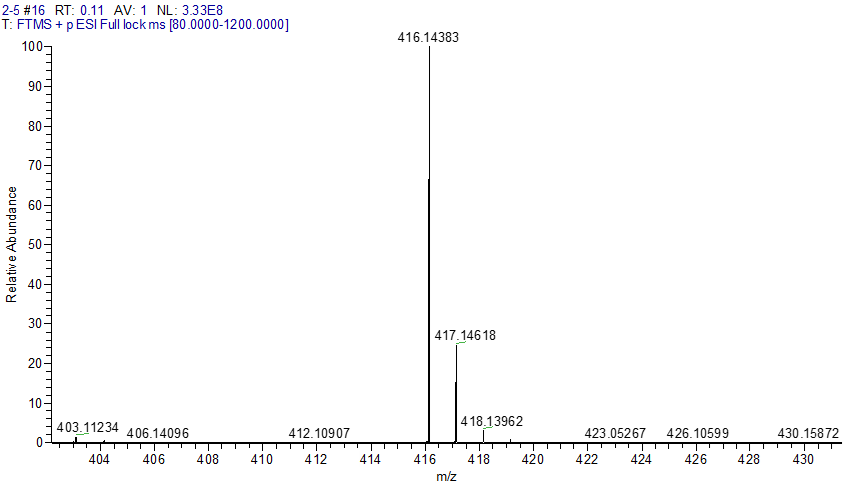


**Supplementary Figure 3:** HRMS of compound SH-337
